# Chronic psychosocial stress and citalopram modulate the expression of the glial proteins GFAP and NDRG2 in the hippocampus

**DOI:** 10.1007/s00213-012-2741-x

**Published:** 2012-05-18

**Authors:** Carolina Araya-Callís, Christoph Hiemke, Nashat Abumaria, Gabriele Flugge

**Affiliations:** 1 Clinical Neurobiology Laboratory, German Primate Center, Leibniz Institute for Primate Research, Kellnerweg 4, 37077 Gottingen, Germany; 2 DFG Research Center for Molecular Physiology of the Brain, Gottingen, Germany; 3 Department of Psychiatry and Psychotherapy, University Medical Center Mainz, Mainz, Germany; 4 Tsinghua-Peking Center for Life Sciences, School of Medicine, Tsinghua University, Beijing, China

**Keywords:** Depression, Glia, Astrocyte, Stress, NDRG2, GFAP, Citalopram

## Abstract

**Rationale:**

It has been suggested that there are causal relationships between alterations in brain glia and major depression.

**Objectives:**

To investigate whether a depressive-like state induces changes in brain astrocytes, we used chronic social stress in male rats, an established preclinical model of depression. Expression of two astrocytic proteins, the intermediate filament component glial fibrillary acidic protein (GFAP) and the cytoplasmic protein N-myc downregulated gene 2 (NDRG2), was analyzed in the hippocampus. For comparison, expression of the neuronal protein syntaxin-1A was also determined.

**Methods:**

Adult male rats were subjected to daily social defeat for 5 weeks and were concomitantly treated with citalopram (30 mg/kg/day, via the drinking water) for 4 weeks.

**Results:**

Western blot analysis showed that the chronic stress downregulated GFAP but upregulated NDRG2 protein. Citalopram did not prevent these stress effects, but the antidepressant per se downregulated GFAP and upregulated NDRG2 in nonstressed rats. In contrast, citalopram prevented the stress-induced upregulation of the neuronal protein syntaxin-1A.

**Conclusions:**

These data suggest that chronic stress and citalopram differentially affect expression of astrocytic genes while the antidepressant drug does not prevent the stress effects. The inverse regulation of the cytoskeletal protein GFAP and the cytoplasmic protein NDRG2 indicates that the cells undergo profound metabolic changes during stress and citalopram treatment. Furthermore, the present findings indicate that a 4-week treatment with citalopram does not restore normal glial function in the hippocampus, although the behavior of the animals was normalized within this treatment period, as reported previously.

## Introduction

Chronic stress is considered a major risk factor for the development of affective disorders such as depression. Taking into consideration the large body of evidence of the role of glial cells in brain homeostasis, modulation of neuronal functioning (including synaptic activity), and adult neurogenesis (Allaman et al. 2011a, b; Seth and Koul 2008), one might assume that glial dysfunction contributes to pathological processes that lead to depression. Several changes in glia have been reported in depressed patients. Immunoreactivity for the astrocytic excitatory amino acid transporter 2 was reduced in the orbitofrontal cortex of patients with major depression (Ongur et al. 1998; Miguel-Hidalgo et al. 2010). Furthermore, lowered immunoreactivity for the glial fibrillary acidic protein (GFAP) was observed in the prefrontal cortex of young depressed subjects (Rajkowska and Miguel-Hidalgo 2007), as well as in the anterior cingulate cortex of depressed patients of different ages (Cotter et al. 2001). However, relatively little is known about presumptive changes in other glial components in relation to depression.

In preclinical research, animal models of chronic stress serve as valuable tools to study the molecular mechanisms that may lead to depression. Among these, social stress in male animals that are defeated daily by a dominant counterpart has been widely used as a paradigm to induce chronic psychological stress (Willner and Mitchell 2002). Social defeat is a strong stressor in males that leads to long-lasting brain and behavioral changes, as determined in several species, including the rat (Blanchard et al. 2001; Buwalda et al. 2005; Koolhaas et al. 2011), mouse (Bartolomucci 2007), and tree shrew (Fuchs and Flugge 2002). The effects of chronic social stress on neurons include retraction of the dendrites of pyramidal cells in the hippocampus (Magarinos et al. 1996), reduced neurogenesis in the subgranular zone of the dentate gyrus (Czeh et al. 2001), and lowered expression of glycoprotein M6 in the axonal membrane of glutamatergic neurons (Alfonso et al. 2004; Cooper et al. 2009). Chronic social stress also affected glia cells in the hippocampus of male tree shrews, where it reduced the number of immunocytochemically detectable astrocytes (Czeh et al. 2006). Using another stress paradigm, it was shown that daily immobilization of rats for 3 weeks upregulates the expression of the glial glutamate transporter 1 (Reagan et al. 2004). Furthermore, a reduction in gliogenesis in the prefrontal cortex (PFC) of rats after chronic unpredictable stress has been reported (Banasr et al. 2007), and Czeh et al. (2007) showed similar effects in the medial PFC after chronic social defeat stress.

To investigate whether chronic social stress in male rats affects astrocytes, we analyzed the expression of GFAP. This cytoskeletal protein is involved in processes related to cell movement and structure and has been proposed to play a role in cell communication such as astrocyte–neuron interactions (Nedergaard et al. 2003). It is strongly expressed in reactive astrocytes (Middeldorp and Hol 2011). Furthermore, we determined NDRG2, a protein expressed in the cytoplasm of astrocytes (Okuda et al. 2008). NDRG2 is encoded by a member of the family of N-myc downregulated genes and has been suggested to be involved in cell proliferation and differentiation and may function as a tumor suppressor (Qu et al. 2002; Deng et al. 2003; Takahashi et al. 2005a; Hu et al. 2006). It is expressed throughout the body, but shows higher levels in the brain, skeletal muscle, liver, and heart (Okuda et al. 2008). Data on the expression of GFAP and NDRG2 after chronic social stress may shed light on the role of astrocytes in depression.

Previous studies have shown that certain effects of chronic stress can be prevented by concomitant treatment with antidepressive drugs; e.g., changes in the expression of the mRNA for synaptic vesicle protein 2b in the dorsal raphe nucleus of rats experiencing daily social defeat were abolished by the selective serotonin reuptake inhibitor (SSRI) citalopram (Abumaria et al., 2007). Among the various SSRIs available, citalopram exhibits one of the highest levels of specificity for 5-HT reuptake inhibition, with only minimal effects on norepinephrine and dopamine uptake (Hiemke and Hartter 2000; Hyttel 1977). The therapeutic effects of SSRIs are thought to be initiated by the rise in extracellular serotonin concentration after initiation of the antidepressive treatment, followed by long-term effects on structural and synaptic neuroplasticity, which are observed only after continued treatment (Duman et al. 1997; Manji et al. 2003; Sairanen et al. 2007). Some studies have analyzed SSRI effects on the expression of neuronal genes that are directly involved either in serotonergic transmission or in cell survival and neuroplasticity (Duman et al. 1997; Manji et al. 2003; Alfonso et al. 2004; Abumaria et al. 2007; Sairanen et al. 2007; Boer et al. 2010; Bethea et al. 2011). However, to date, little is known about the regulation of glial genes by antidepressants in preclinical models of depression.

Therefore, the present study aimed to investigate the effects of chronic social stress and citalopram treatment on the expression of the glial genes *gfap* and *ndrg2* in the hippocampal formation, and to discuss possible implications of presumptive effects for the pathophysiology of depression. Expression of the synaptic protein syntaxin-1A was also analyzed to find out whether there might be differences in the stress and antidepressant regulation of a neuronal versus the astrocytic proteins.

## Materials and methods

### Experimental animals

Male Wistar rats (Harlan Winkelmann, Borchen, Germany) weighing 180–200 g on arrival were housed individually in type II Makrolon cages with food and water ad libitum. The animal facility had a reversed 12:12 h light/dark cycle (lights off at 7:00 a.m.) and the temperature was maintained at 21 °C. The experiments were conducted during the dark phase, which corresponds to the active phase of the rats, and all experimental manipulations were performed under dim red light. Lister Hooded male rats weighing 300–350 g (Harlan Winkelmann, Borchen, Germany) were used as residents. These males were paired with sterilized females and housed in large plastic cages (60 × 40 × 40 cm = l × w × h) situated in a separate room from that used to house the Wistar rats. After arrival, animals were habituated to the conditions for 2 weeks and were handled daily. All animal experiments were in accordance with the directive of November 24, 1986 of the Council of the European Communities (86/609/ECC), including Position 6106/20 of the EU Council of May 26, 2010, and were approved by the Government of Lower Saxony, Germany.

### Chronic social stress

In a pilot experiment, we first determined whether chronic social stress may have an impact on expression of NDRG2 in the hippocampus. For this we used eight controls and eight animals that experienced daily social defeat using a resident–intruder paradigm according to an established protocol (Rygula et al. 2005). This paradigm is based on the social subordination induced by the aggressive territorial exclusion of unfamiliar males (intruders) by a resident dominant male rat with prolonged periods of daily social defeat (Blanchard et al. 2001). After a habituation period of 2 weeks when the rats were daily handled and weighed, the animals were submitted to daily social defeat for 4 weeks. The procedure is as follows: before the daily social confrontation, the female is removed from the cage of the resident male. Subsequently, the intruder is introduced into the resident's cage. During the first 1–3 min of confrontation, the resident attacks and defeats the intruder, which displays a defensive behavior characterized by submissive posture, freezing behavior, and/or flight. Immediately thereafter, the intruder is placed in a small wire-mesh cage within the resident's home cage for 1 h. In this way, the intruder is protected from further physical injuries, but remains in olfactory, visual, and auditory contact with the resident. Subsequently, the intruder is returned to its home cage. This procedure is performed every day at 9:00 a.m. (during the activity period). Control animals are handled daily during the social stress period.

**Fig. 1:**
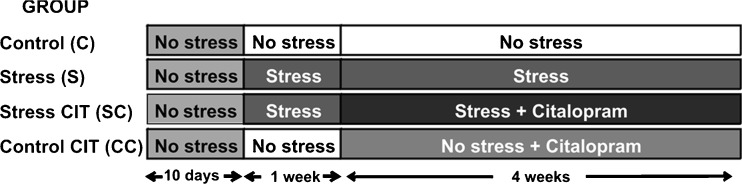
Design of the chronic stress and chronic citalopram experiment. Four groups of male Wistar rats were analyzed, control, stress, control CIT, and stress CIT. After 1 week of habituation (no stress), stress animals were subjected to daily social defeat for 5 weeks. Animals in the control CIT group received the drug during 4 weeks, daily via the drinking water. Animals in the stress CIT group also received the drug during 4 weeks while the daily stress exposure was continued

In the subsequent experiment with chronic stress and chronic citalopram four experimental groups were used: control (C), stress (S), control citalopram (CC), and stress citalopram (SC) (*n* = 15 for groups C and S; *n* = 10 for groups CC and SC; Fig. 1). After a habituation period of 10 days, daily psychosocial stress was induced using the above resident–intruder paradigm (Rygula et al. 2005). Body weight was recorded throughout the entire experimental period: During the habituation period, body weight was measured three times (on days 2, 5, and 10); subsequently, it was recorded daily. Body weight gain was calculated as a percentage of the individual baseline body weight at the beginning of the experiment. The animals were either decapitated (*n* = 10 per group) or perfused (*n* = 5 for groups C and S), 24 h after the last stress exposure (see below). After decapitation, the adrenal glands were removed and weighed; weight was expressed as a percentage of body weight.

### Citalopram administration and determination of the parent drug and its metabolites

CIT hydrochloride was kindly provided by Lundbeck (A/S, Copenhagen, Denmark). The drug was administered orally via the drinking water, as described, to minimize the stress effects that may be induced by injection; the amount of consumed water was measured daily by weighing the bottles. As in previous experiments, animals were treated chronically with CIT (30 mg/kg) for 4 weeks, starting 1 week after the beginning of the social stress, whereas untreated animals received tap water (Fig. 1; Rygula et al. 2006; Abumaria et al. 2007). The concentration of drug was adjusted (taking into account body weight and consumed water) and dissolved in the average volume of consumed water (approximately 30 mL/day). The solution containing the drug was prepared freshly every third day and poured into light-protected bottles. The bottles were weighed (always at 9:00 a.m.) after preparation and 24 h later to monitor drug intake. At the end of the experimental period, animals were decapitated and trunk blood and brain tissue (neocortex) were collected for the analysis of CIT, desmethylcitalopram (DCIT), and didesmethylcitalopram (DDCIT). Samples were stored at −20 °C and light protected until assayed for the drug and its metabolites. Analysis of CIT, DCIT, and DDCIT was performed using HPLC (Abumaria et al. 2007). Data are expressed as nanograms per milliliter for blood samples and nanograms per gram of tissue for neocortex samples. To compare blood and cortex values, the ratio of CIT/DCIT was calculated.

### Perfusion and brain tissue preparation

#### Perfusion

For fixation of the brains, animals were deeply anesthetized with a mixture of xylazine (50 mg/mL), ketamine (10 mg/mL), and atropine (0.1 mg/mL). Subsequently, the animals were transcardially perfused with cold (4 °C) 0.9 % NaCl for 3 min, followed by cold (4 °C) 4 % paraformaldehyde (PFA) in 0.1 M phosphate buffer (pH 7.2) for approximately 12 min. The heads were postfixed in fresh 4 % PFA at 4 °C overnight. The following day, brains were removed from the crania and immersed in 30 % sucrose in phosphate-buffered saline (PBS) (0.137 M NaCl, 2.7 mM KCl, 4.3 mM Na_2_HPO_4_ × 12H_2_O, and 1.4 mM KH_2_PO_4_; pH = 7.2) and incubated at 4 °C for 2 days. The brain blocks were then frozen on dry ice and stored at −80 °C until coronal sectioning was performed on a Leica cryostat (CM3050S) at a thickness of 40 μm (−1.80 to −6.04 mm Bregma; Paxinos and Watson 1986).

#### Dissection of the hippocampus for Western blot analysis

Ten animals from each group were used for protein analysis. Immediately after decapitation, the brains were quickly removed from the crania. After removal of the cerebellum and brain stem, a cut was performed between the cortical hemispheres, down to the corpus callosum. The hemispheres were carefully shifted apart and the hippocampal formation was removed using a sterile spatula. Both hippocampi (left and right) were collected, immediately frozen in liquid nitrogen, and stored at −80 °C for subsequent use in molecular analyses.

### Immunohistochemistry

#### Immunohistochemistry for light microscopy

From a total of ten animals (five from group C and five from group S), eight floating sections per animal were immunostained and analyzed. The sections were processed in parallel to avoid variations in staining intensity. Sections were washed and were then incubated for 30 min in 1 % H_2_O_2_ in PBS to inactivate endogenous peroxidase activity. All washing steps consisted of three washes of 10 min each in PBS. After washing, sections were incubated in blocking solution (3 % normal goat serum (NGS; Vector Laboratories, Burlingame, CA, USA) in PBS containing 0.3 % Triton X-100) for 1 h to avoid nonspecific binding of the antibody. Subsequently, sections were incubated overnight at 4 °C with a mouse polyclonal anti-NDRG2 antibody (Abnova, Taiwan; dilution, 1:500) in PBS/NGS/T (1 % NGS and 0.3 % Triton X-100). After washing, the sections were incubated with a biotinylated goat anti-mouse IgG (Vector Laboratories; dilution, 1:400) in PBS/NGS/T for 2 h. Subsequently, the sections were washed and incubated in the ABC complex (ABC Kit, Vector Laboratories) in PBS containing 3 % NGS for 1.5 h. Finally, sections were incubated in 0.025 % 3,3′-diaminobenzidine (DAB, Peroxidase Substrate Kit, Vector Laboratories) with 0.01 % H_2_O_2_. The sections were then washed, mounted on glass slides, and left to dry overnight at 37 °C. The following day, sections were counterstained with methyl green, cleared in xylene, and cover slipped using Eukitt mounting medium. Sections were examined with the Axiophot II microscope using a Plan-Apochromat ×63 oil immersion objective (NA 1.4; ZEISS, Carl Zeiss MicroImaging GmbH, Göttingen, Germany).

#### Immunohistochemistry for confocal microscopy

Double labeling experiments with immunofluorescent secondary antibodies were performed to visualize NDRG2 together with GFAP, or together with neuron-specific enolase (NSE) in sections from the hippocampus (see antibody list in Table 1). For each experiment, ten brain sections from the level approximately −3.6 mm from Bregma (Paxinos and Watson, 1986) were washed in PBS, three times, 10 min each. Sections were then incubated in blocking solution (3 % NGS and 3 % normal horse serum, NHS, Vector Laboratories Burlingame) in PBS containing 0.3 % Triton X-100) for 1 h to avoid nonspecific binding of the antibody. Subsequently, for the NDRG2/GFAP labeling, sections were incubated overnight at 4 °C with a mouse polyclonal anti-NDRG2 antibody (Abnova, Taiwan; dilution, 1:500) and a rabbit polyclonal anti-GFAP antibody (Synaptic Systems, SySy, Gottingen; dilution, 1:5,000) in PBS/NGS/NHS/T (3 % NGS, 3 % NHS, and 0.3 % Triton X-100). After washing, sections were incubated for 2 h with Alexa 488- and 594-coupled secondary antibodies (Invitrogen/Molecular Probes, Carlsbad, CA, USA; dilution, 1:500) in PBS/NGS/NHS/T. For the NDRG2/NSE labeling, sections were incubated overnight at 4 °C with the anti-NDRG2 antibody and rabbit polyclonal anti-NSE antibody (AB951, Chemicon International, dilution 1:2,000) in PBS/NGS/NHS/T (3 % NGS, 3 % NHS, and 0.3 % Triton X-100). After washing, sections were incubated for 2 h with Alexa 488- and 594-coupled secondary antibodies (Invitrogen/Molecular Probes) in PBS/NGS/NHS/T. Finally, sections were rinsed in PBS and briefly in bidistilled H_2_O before mounting. Microscopy was performed using a LSM Pascal 5 laser scanning confocal microscope (ZEISS). Images were obtained with a Plan-Apochromat ×20x (NA 0.75) or a Plan-Apochromat ×63 oil immersion objective (NA 1.4; ZEISS).

**Table 1:** Antibodies and incubation conditions for the quantitative Western blots

Antibody	Company	Working dilution	Incubation
GFAP (rabbit polyclonal)	SYSY	1:4,000	ON/4 °C
NDRG2 (mouse polyclonal)	Abnova	1:4,000	ON/4 °C
Neuron specific enolase (NSE)	Chemicon International	1:2,000	ON/4 °C
Syntaxin-1A (mouse monoclonal)	SYSY	1:1,000	ON/4 °C
ß-actin (mouse monoclonal)	Sigma	1:8,000	30 min/RT
Peroxidase-conjugated goat anti-rabbit	DakoCytomation	1:8,000	1 h/RT
Peroxidase-conjugated goat anti-mouse	Santa Cruz Biotechnology	1:4,000	1 h/RT

*RT* room temperature, *ON* overnight

### Counting NDRG2-positive cells in the hippocampus

The quantification of NDRG2-immunoreactive cells in the stratum oriens (SO) and stratum radiatum (SR) of the CA1 region in the hippocampus was performed using a Zeiss III microscope (Carl Zeiss, Oberkochen, Germany) and the Neurolucida system (Microbrightfield Inc., Williston, USA, Version 7).

In the SO and SR, cells were counted in delineated areas of approximately 90,000 and 185,000 μm^2^, respectively (Fig. 6). Data are reported as density (cells per square micrometer) multiplied by 1,000. Data from both hemispheres were collected separately to assess possible hemispheric effects of chronic stress.

### Quantitative immunoblotting

Samples of hippocampi were homogenized with a dounce homogenizer (tight pestle) in ice-cold homogenization buffer (150 mM NaCl, 1 mM Tris/HCl, pH 8.0), 7 % glycerol, and 0.1 % Triton X-100) containing protease inhibitors (Complete Protease Inhibitor Cocktail Tablets, Roche Diagnostics). The protein homogenates were centrifuged for 5 min at 13,000 rpm and supernatants were recovered; aliquots were stored at −80 °C. Protein concentrations were measured using the Bio-Rad DC Protein assay (Bio-Rad Laboratories, Hercules, USA).

For electrophoresis, the protein extracts of hippocampi were denatured for 10 min at 70 °C in Laemmli buffer with dithiothreitol and chilled on ice for 5 min. Samples were loaded and electrophoretically separated in 12.5 % SDS gels under reducing conditions. Subsequently, proteins were transferred from the gel to a nitrocellulose membrane (Schleicher & Schuell, Dassel, Germany) via semidry electroblotting for 2 h at 1 mA/cm^2^ in transfer buffer containing 25 mM Tris base (pH 8.3), 150 mM glycine, and 10 % methanol. The membrane was blocked for 1 h with 5 % milk powder in PBS, followed by incubation with the primary antibody. Antibody concentrations and incubation times were adjusted to yield visible bands of the proteins of interest (Table 1). After incubation with the primary antibodies, the membranes were carefully washed with PBS-T (0.1 % Tween-20 in PBS) and incubated with the peroxidase-labeled secondary antibodies. After 1 h of incubation at RT, the membranes were washed three times with PBS-T and three times with PBS. Finally, the membranes were subjected to enhanced chemoluminescence amplification and the bands were visualized on Hyperfilm^TM^.

Subsequently, to visualize ß-actin as a reference protein, membranes were stripped in S-PBS (PBS, 2 % SDS, and 0.7 % ß-mercaptoethanol) for 2 h, and incubated with a monoclonal anti ß-actin antibody, followed by the corresponding secondary antibody. The relative optical density of the bands was quantified and analyzed using the ImageJ software (Rasband, W.S., ImageJ, U.S. National Institutes of Health, Bethesda, Maryland, USA, http://imagej.nih.gov/ij/, 1997–2011). Data are presented as a percentage of the mean value from the control group.

### Statistical analyses

All data were tested for normality (95 % confidence interval); results are expressed as means ± SEM. Significance was set at a probability level of 95 % (*P <* 0.05). Statistical analyses and comparisons were performed using (1) the two-tailed unpaired Student’s *t* test, or (2) one-way analysis of variance (ANOVA) followed by Bonferroni’s post hoc test to depict differences between the groups in the chronic stress and citalopram treatment experiment, and (3) two-way ANOVA to examine main effects of the factors ‘stress’ and ‘drug’ in the chronic stress and citalopram treatment experiment (SigmaPlot 12.0). Body weight was determined at the end of each experimental week, and body weight gain was expressed as a percentage of baseline. These data were subjected to two-way ANOVA for repeated measures (data from the four groups, determined in each experimental week; SigmaPlot 12.0). Graphs were generated with GraphPad 4.0 (GraphPad Software, Inc., San Diego, CA, USA). Values that were two standard deviations higher or lower than the mean were excluded from the analyses.

## Results

### Effects of chronic stress and citalopram on body and adrenal weight

**Fig. 2:**
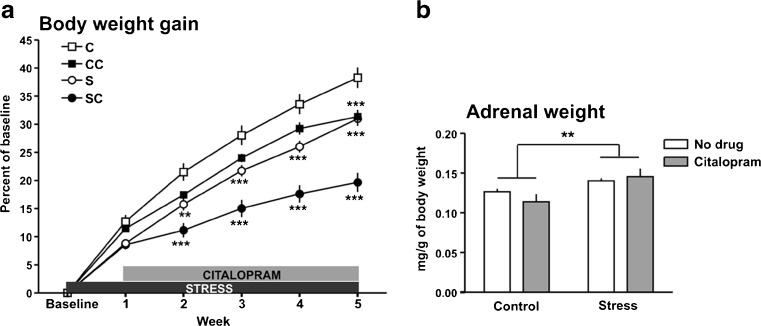
Effects of chronic psychosocial stress on body weight gain and adrenal weight. **a** Chronic social defeat reduced body weight gain in stressed rats compared to control. Weekly data are expressed as percentages of body weight at the beginning of the experiment (mean ± SEM); **P < 0.01, ***P < 0.001 significant difference to control as determined by two-way repeated measures ANOVA with Bonferroni’s post hoc test. **b** Stress also caused a significant increase in adrenal gland weight; **P < 0.01, significant difference between all controls (including control CIT) and all stressed animals (including stress CIT) determined with the *t* test. *C* control, *S* stress, *SC* stress CIT, *CC* control CIT

Five weeks of daily social defeat caused a reduction in body weight gain in the stressed rats (Fig. 2a). Two-way repeated measures ANOVA with Bonferroni’s post hoc test revealed significant differences between stress and control from the second stress week onwards. Stressed animals treated with the drug gained less weight compared to control from the second stress week onwards. In control animals, citalopram affected body weight gain only in the fifth week. There was a significant effect of the treatments (*F*_(3, 230)_ = 15.72, *P* < 0.0001), of weeks (*F*_(5,230)_ = 1069, *P* < 0.0001), and an interaction (*F*_(15,230)_ = 18.98, *P* < 0.0001).

Adrenal weight (milligrams per gram of body weight) was increased in the stressed animals (Fig. 2b). Two-way ANOVA showed a significant stress effect (*F*_(1,32)_ = 11.31, *P* < 0.01), but no effect of the drug and no interaction. The *t* test performed with all control and all stressed animals (including those treated with citalopram) revealed a significant difference between these two groups (*P* < 0.01; Fig. 2b).

### Serum and brain tissue concentration of citalopram and its metabolites

CIT, DCIT, and DDCIT were detectable in the blood and brain (Table 2). Blood concentrations were in the range of those reported previously in a study using the same citalopram dose (Abumaria et al. 2007). No significant differences were observed between controls and stressed animals (Table 2). In plasma, the average ratio of CIT/DCIT was 0.55, and the ratio of CIT/DDCIT was 0.46. In the cortex, the ratio of CIT/DCIT was 2.10, and the ratio of CIT/DDCIT was 2.96. The accumulation of metabolites in the brain was less pronounced than that of the parent drug.

**Table 2:** Citalopram and its metabolites in blood and neocortex

Compound	Control citalopram		Stress citalopram	
	Blood (ng/mL)	Brain (ng/g)	Blood (ng/mL)	Brain (ng/g)
CIT	99.9 ± 14.5	674.0 ± 137.3	84.3 ± 8.9	535.0 ± 86.6
DCIT	180.5 ± 17.5	301.5 ± 21.0	191.4 ± 18.5	339.0 ± 29.6
DDCIT	231.2 ± 8.5	226.0 ± 9.2	243.5 ± 16.8	228.0 ± 16.1

Data are presented as mean ± SEM

### Chronic psychosocial stress increases NDRG2 protein in the hippocampus

**Fig. 3:**
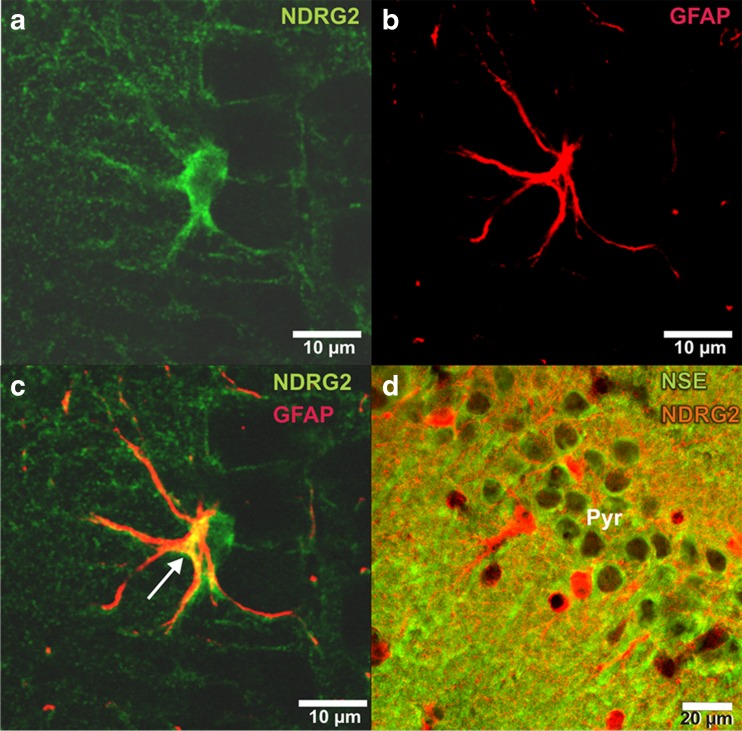
NDRG2 colocalizes with GFAP but not with the neuronal marker protein NSE. **a** NDRG2 immunofluorescence (*green*) in an astrocyte in the stratum radiatum of the hippocampus. Please note that NDRG2 is found throughout the cytoplasm of the astrocyte. **b** GFAP immunofluorescence (*red*) in the same astrocyte as in A. **c** Merged picture showing NDRG2-GFAP colocalization (*yellow*) in the cytoplasm of the astrocyte (*arrow*). **d** Double immunofluorescence for NDRG2 (*red*) and NSE (*green*) reveals no colocalization in any neuron

It has been reported previously that NDRG2 is expressed in astrocytes (Okuda et al. 2008). The present double immunofluorescence experiment confirmed this showing that colocalization with the intermediate filament protein GFAP was only found in the cytoplasm of the astrocyte (Fig. 3a–c). In contrast, there was no NDRG2 in cells which were labeled with the antibody against the neuronal marker NSE (Fig. 3d).

In a pilot experiment with control and stressed rats, we analyzed hippocampal NDRG2 expression by Western blotting (ß-actin as reference protein). We found that 4 weeks of daily social defeat increased NDRG2 protein in the hippocampus (data were expressed as a percentage of the mean control value: control, 100.0 ± 18.3; stress, 197.9 ± 32.79; *n* = 8/group; *t* test: *P* < 0.05). Thereafter, the experiment with chronic social stress and citalopram treatment was performed.

### Effects of chronic psychosocial stress on GFAP and NDRG2 in the hippocampus: no reversal by citalopram

**Fig. 4:**
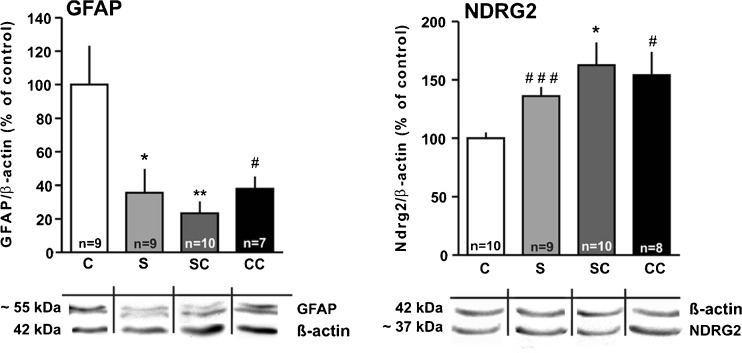
Effects of chronic psychosocial stress and concomitant citalopram treatment on GFAP and NDRG2 expression in the hippocampus (Western blot analysis; β-actin as reference protein). Representative blotting membranes are shown *beneath the bar graphs*. Results are presented as percentages of the mean control value (mean ± SEM). Statistical differences resulting from one-way ANOVA with Tukey’s multiple comparison test: *P < 0.05, **P < 0.01 vs. control. Statistical difference to control as determined with the *t* test: ^#^ *P* < 0.05; ^###^ *P* < 0.001. *C* control, *S* stress, *SC* stress citalopram, *CC* control citalopram

In the experiment with four groups of rats (control, stress, control citalopram, and stress citalopram), GFAP was downregulated by chronic stress, and citalopram did not prevent this effect (Fig. 4). The Western blot displayed two GFAP bands, one at ~55 kDa (representing the intact molecule) and one at 48 kDa (representing a proteolytic cleavage product) (Straccia et al. 2011). To determine protein expression, the optical density of the two bands was analyzed. Two-way ANOVA showed a significant stress effect (*F*_(1, 31)_ = 11.28, *P* < 0.01) and an effect of citalopram (*F*_(1, 31)_ = 4.47, *P* < 0.05), but no interaction.

**Fig. 5:**
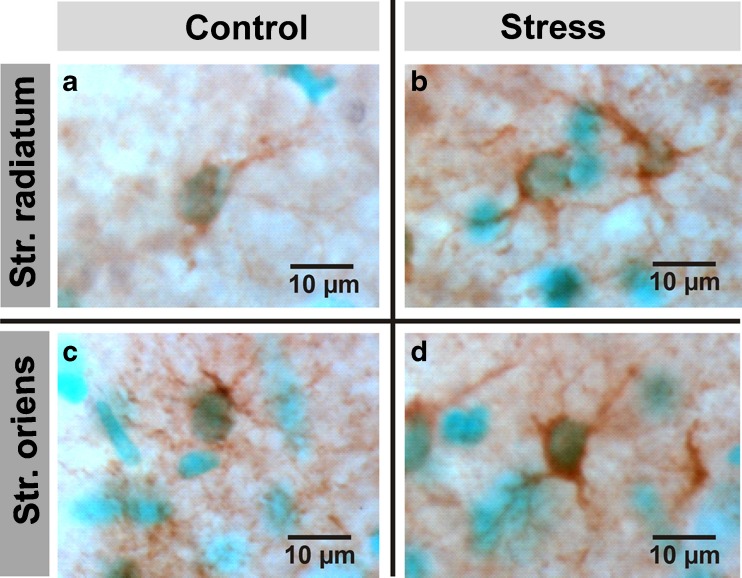
Representative images of NDRG2-immunoreactive astrocytes in hippocampal regions stratum radiatum and stratum oriens, in a control (**a**, **c**) and a stressed rat (**b**, **d**)

NDRG2 expression appeared slightly increased by stress, but two-way ANOVA revealed only an effect of the drug (*F*_(1,33)_ = 7.74, *P <* 0.01; Fig. 4). Accordingly, one-way ANOVA with Bonferroni’s post hoc test revealed no significant difference between stress and control. Only when control and stress were compared with the *t* test a difference was observed (*P* < 0.001). However, NDRG2 in the stress CIT group was significantly increased as compared to control (one-way ANOVA with post hoc test, *P <* 0.05; Fig. 4). Figure 5 shows NDRG2-immunoreactive astrocytes in controls and stressed rats.

**Fig. 6:**
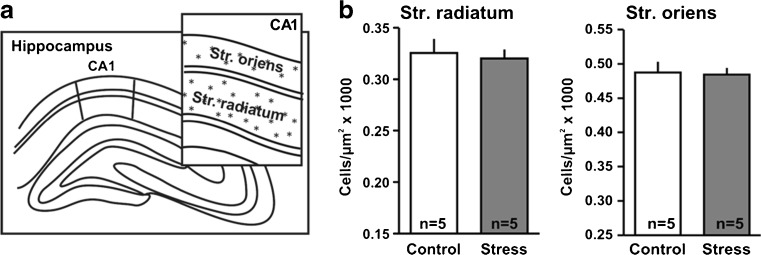
Quantification of NDRG2-positive cells in hippocampal region CA1. **a** Schematic illustrating that labeled cells (*asterisks*) were counted in areas of approximately 90,000 μm^2^ in the stratum oriens and 185,000 μm^2^ in the stratum radiatum. **b** No significant differences with respect to the densities of NDRG2-positive cells in stress and control were observed. Results are presented as numbers of immunopositive cells/μm^2^, multiplied by 1,000 (mean ± SEM)

We tested whether the upregulation of NDRG2 in the hippocampus was due to an increased number of cells that express this protein. NDRG2-positive astrocytes were counted in region CA1 of the hippocampus, in stratum radiatum and stratum oriens. This quantification of NDRG2-positive astrocytes (number of cells per square micrometer multiplied by a factor of 1,000) revealed no significant stress-induced change in cell density (Fig. 6).

### Chronic psychosocial stress upregulates syntaxin-1A in the hippocampus: reversal by citalopram

**Fig. 7:**
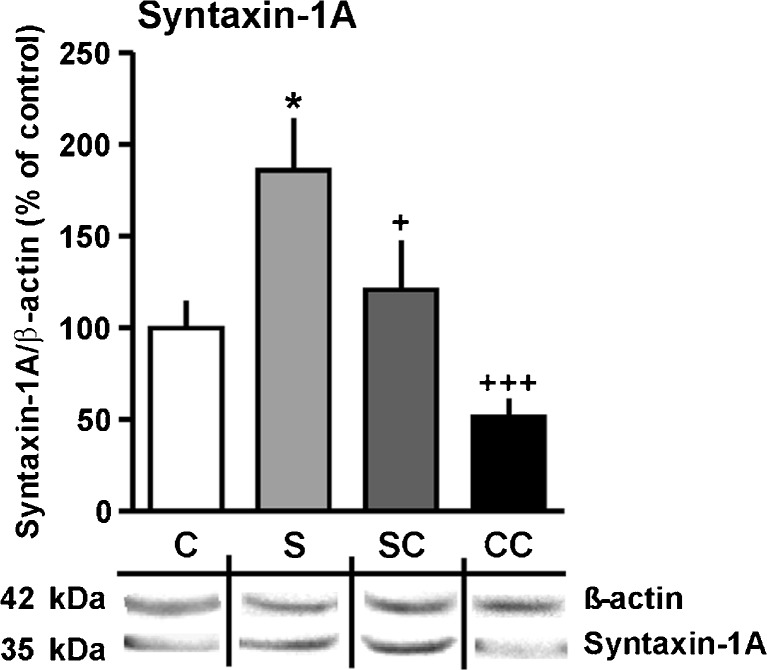
Effects of chronic psychosocial stress and concomitant citalopram treatment on syntaxin-1A expression in the hippocampus (Western blot analysis; β-actin as reference protein). Representative blotting membranes are shown *beneath the bar graph*. Results are presented as percentages of the mean control value (mean ± SEM). Statistical differences resulting from one-way with Tukey’s multiple comparison test: *P < 0.05 vs. control; ^+^ *P* < 0.05, ^+++^ *P* < 0.001, vs. stress. *C* control, *S* stress, *SC* stress CIT, *CC* control CIT

Quantification of the synaptic protein syntaxin-1A was performed by Western blotting using ß-actin as an internal reference to normalize the data (Fig. 7). The blot showed the expected band in the molecular range of 35 kDa. Two-way ANOVA revealed effects of stress (*F*_(1,32)_ = 13.03, *P <* 0.001) and citalopram (*F*_(1,32)_ = 6.98, *P <* 0.05), but no interaction. One-way ANOVA with Bonferroni’s post hoc test showed that syntaxin-1A was upregulated by stress (*P <* 0.05, compared to control) and normalized by citalopram (*P <* 0.05 compared to stress). There was no significant difference between control and control CIT, but a significant difference between stress and control CIT (*P <* 0.001).

## Discussion

We investigated the effects of chronic social stress and antidepressant treatment on the expression of astrocytic proteins. Five weeks of daily social confrontation downregulated GFAP in the hippocampus of the male rats, and there was a trend of a stress-induced NDRG2 upregulation. However, contrary to what we had expected, these stress effects were not reversed by chronic citalopram although a previous study had shown that the SSRI normalizes depressive-like behavior. Citalopram only prevented the stress-induced upregulation of the neuronal protein syntaxin-1A. These findings indicate that chronic social stress leads to profound changes in astrocytes, and that normalization of the expression of the astrocytic genes in the hippocampus is not a prerequisite for the therapeutic actions of citalopram.

### Glial changes and depression

It has been hypothesized that glial dysfunction plays a key role in the pathophysiology of psychiatric disorders, including major depression (Banasr et al. 2007). However, it is still unknown which presumptive glial changes are causal for depression, although some post mortem data from patients indicated alterations in astrocytes: GFAP immunoreactivity in cell bodies and fibers was significantly reduced in hippocampal regions CA1 and CA2 of patients with major depression (MD), as well as in patients treated chronically with synthetic glucocorticoids (Muller et al. 2001). GFAP immunoreactivity was also reduced in the left orbitofrontal cortex of MD patients (Miguel-Hidalgo et al. 2010). Moreover, there is evidence that neurons and glia cells in the hippocampus of MD patients are more densely packed than in healthy controls (Stockmeier et al. 2004). The present data derived from the preclinical model of depression confirm that in a depressive-like state, GFAP is reduced in the hippocampal formation and there is evidence for profound metabolic changes in the astrocytes.

### Chronic stress and GFAP

We used chronic social stress in rats to provoke central nervous changes that resemble symptoms of depression, because stressful life events can lead to depressive disorders (Kendler et al. 2004; McEwen et al. 2002). As expected and shown previously, the chronic stress reduced body weight gain in the intruder animals and increased the weight of adrenal glands which indicates hyperactivity of the hypothalamic–pituitary–adrenal axis (Abumaria et al. 2007; Czeh et al. 2006; Rygula et al. 2005).

Immunocytochemistry with anti-GFAP antibodies is a widely used tool to visualize presumptive pathological changes in brain glia, in particular changes in reactive astrocytes (Middeldorp and Hol 2011). Several studies showed that GFAP expression is regulated by stress: acute systemic heat stress (38 °C for 4 h) and running-wheel activity increased GFAP immunoreactivity in several brain regions and in the hippocampus, respectively, possibly indicating the stimulation of reactive astrocytes (Lambert et al. 2000; Sharma et al. 1992). In contrast, juvenile separation stress diminished GFAP-immunoreactive astrocytes in the medial prefrontal cortex of degus (Braun et al. 2009), and another strong psychological stress, chronic social defeat, reduced the number of GFAP-immunoreactive cells in the hippocampus of male tree shrews (Czeh et al. 2006). Furthermore, chronic mild stress in rats lowered GFAP mRNA in the rat neocortex (Banasr et al. 2010). Coinciding with this, the present data reveal reduced GFAP in the hippocampus of male rats after chronic social defeat. Stress-induced elevations of glucocorticoids may have contributed to this effect as corticosterone lowered GFAP levels in the rat brain (Nichols et al. 1990; O'Callaghan et al. 1991).

Some authors interpreted stress-induced reductions in GFAP immunoreactivity as glial atrophy (Liu et al. 2011). However, data from the hippocampal formation reveal no experimental evidence for atrophy processes, including apoptosis. Apoptosis in the hippocampus is a rather rare event, and chronic social stress increased apoptosis only in the hilus, but even reduced the number of apoptotic cells in other hippocampal subfields (Lucassen et al. 2001). In addition, since GFAP-immunoreactive structures constitute only ~15 % of the total astrocytic volume, the reduction in the protein should not be taken as a measure of cell numbers (Bushong et al. 2002; Lavialle et al. 2011). Therefore, it is unlikely that the present stress-induced decrease in GFAP accounts for a loss of astrocytes. The low GFAP levels may rather reflect the presence of resting astrocytes because, vice versa, reactive astrocytes show high GFAP expression (Middeldorp and Hol 2011).

### NDRG2 and stress

Our present data confirm that NDRG2 is expressed in astrocytes but not in neurons. *NDRG2* is among the genes that are regulated by steroid hormones (Nichols and Finch 1994; Takahashi et al. 2012). Corticosterone upregulated NDRG2 mRNA in the adult rat heart and cerebral cortex, as well as in cultured astrocytes (Nichols 2003). Accordingly, as the hippocampus is a major target of circulating glucocorticoids, one may interpret the upregulation in the hippocampal formation as a direct effect of elevated glucocorticoids. However, glucocorticoid receptors are present in almost all brain regions, and NDRG2 transcripts were also upregulated in the dorsal raphe nucleus of male rats after chronic social stress (Abumaria et al. 2006). A further link between glucocorticoid action and NDRG2 is provided via serum- and glucocorticoid-induced kinase 1 which phosphorylates this protein (Murray et al. 2004; Takahashi et al. 2012).

Several former studies tried to elucidate the role of NDRG2 in cell proliferation and differentiation, respectively. NDRG2 expression increases during the late stages of embryogenesis as shown in mice, which suggested an involvement in tissue differentiation and maintenance (Hu et al. 2006). In vitro, NDRG2 mRNA increased in the course of nerve growth factor-evoked differentiation in neuronal PC12 cells (Takahashi et al. 2005a). In the adult brain, the protein is found in many regions, with particularly strong expression in the subgranular zone of the dentate gyrus, an area of ongoing neurogenesis (Nichols 2003). However, the exact role of NDRG2 in differentiation vs. proliferation of cells is so far unclear: A lack of NDRG2 slowed myoblast proliferation in skeletal muscle tissue (Foletta et al. 2009), but *Ndrg2* gene silencing stimulated proliferation of cultured astrocytes (Takeichi et al. 2011). Interestingly, a recent study indicated that NDRG2 might play a role in immune function: High NDRG2 expression in dendritic cells correlated with suppression of interleukin 10 (IL-10) secretion (Choi et al. 2010). Increased levels of the anti-inflammatory cytokine IL-10 in the blood of women have been associated with states of anger and sadness, although not with stress (Danielson et al. 2011). Nonetheless, it remains to be elucidated whether NDRG2 expression is related to immune functions.

Furthermore, it is interesting to note that NDRG2 may interact with a subunit of Na^+^/K^+^-ATPase as shown in epithelial cells (Li et al. 2011). In astrocytes, Na^+^/K^+^-ATPase is activated after synaptic activity to restore the ion gradient necessary to drive glutamate transport (Haydon and Carmignoto 2006; Pellerin and Magistretti 1997). Astrocytic uptake of glutamate that has been released from neurons plays an especially important role in the hippocampus of stressed subjects where glutamatergic transmission is enhanced (Piet et al. 2004; Popoli et al. 2011).

### Stress-induced changes in astrocytes

The observed stress effects may reflect a change in the metabolic activity of the astrocytes, presumptively a reduction in functions which characterize reactive astrocytes. As astrocytes are dynamic regulators of synaptogenesis and synaptic strength, and control neurogenesis in the adult dentate gyrus, the stress effects observed here may negatively influence neuronal functioning, possibly because of alterations in the capacity of supplying neurotrophic factors to the neuropil (Araque et al. 1999; Goldman 2003; Reuss and Unsicker 2005). However, it remains to be investigated whether the changes in the astrocytes are pathological or adaptive processes that have the potential to protect the neural network.

### Effects of citalopram

We treated the rats orally with citalopram as described before (Rygula et al. 2006). At the end of the chronic treatment, concentrations of the drug and its metabolites in the plasma were in the range of those described before, and concentrations in the cerebral cortex showed that the SSRI reached the brain effectively (Abumaria et al. 2007). Moreover, citalopram reduced body weight gain, which is in accordance with previous observations (D'Souza et al. 2004; Landry et al. 2005). The drug induced no changes in adrenal weight, as described also for other SSRIs (Czeh et al. 2006; Schulte-Herbruggen et al. 2009).

Surprisingly, citalopram did not prevent the stress effects on either GFAP or NDRG2 in the hippocampus. A previous study had shown that also the SSRI fluoxetine did not fully restore the normal features of GFAP-immunoreactive cells in the hippocampus of chronically stressed tree shrews (Czeh et al. 2006). These findings are in contrast with observations regarding the dorsal raphe nucleus (DRN) of rats experiencing chronic social stress, where citalopram normalized *Ndrg2* mRNA expression (Abumaria et al. 2007). One may speculate that the SSRI is more effective in regulating astrocytic gene expression in the DRN, the site of serotonergic neurons, where serotonin transporter blockade probably produces a considerable rise in extracellular 5-HT. Furthermore, the regional difference in the effects of citalopram in the brain may also be related to differences in cellular properties of astrocytes which do not represent a homogeneous cell population (Himeda et al. 2006; Jinno 2011; Kettenmann and Verkhratsky 2008; Wallraff et al. 2004; Walz and Lang 1998).

In contrast to the present data, tianeptine (an atypical antidepressive drug) prevented the upregulation of the glial glutamate transporter 1 in the hippocampus of rats that experienced daily immobilization stress (Reagan et al. 2004). The discrepancy between the present results and those earlier findings may be of course due to the different drugs, but also to the particular stress paradigms. In a chronic mild-stress model, the tricyclic antidepressant clomipramine prevented the downregulation of hippocampal GFAP (Liu et al. 2009).

Citalopram downregulated GFAP and upregulated NDRG2 in the hippocampus of nonstressed rats. Data from mouse astrocytic primary cultures have shown that other antidepressants (amitryptyline, clomipramine) suppressed GFAP promotor activity (Cho et al., 2010). It has been proposed that SSRIs regulate the expression of astrocytic genes via mechanisms that do not necessarily involve serotonin, as discussed for the effects of fluoxetine on S100ß (Tramontina et al. 2008). Fluoxetine upregulated brain-derived neurotrophic factor and increased glucose utilization as well as lactate release in cultured cortical astrocytes (Allaman et al. 2011a, b). In vitro, citalopram even directly evoked calcium signals in astrocytes (Schipke et al. 2011). However, whether similar regulatory mechanisms are responsible for the citalopram effects on GFAP in the hippocampus of the unstressed rats is not known. Contrary to our data on the hippocampus, NDRG2 was found to be downregulated by antidepressants (sertraline, imipramine) in the prefrontal cortex of unstressed rats (Takahashi et al. 2005b).

The effects of citalopram on GFAP and NDRG2 expression observed here go in the same directions as the chronic stress effects. Nevertheless, the underlying mechanisms may be parts of different cellular processes which are so far unknown. In general, the present findings agree with the hypothesis that long-term antidepressive drug treatments stimulate the plasticity of brain cells (Sairanen et al. 2007). This and previous publications have shown that drug-induced plasticity implies a reversal of at least some of the stress/depression-related changes. However, the present data provide no evidence that citalopram reverses stress effects on astrocytes in the hippocampus.

## Conclusions

The downregulation of GFAP in the hippocampus induced by chronic social stress suggests the presence of resting astrocytes. This, together with the moderate upregulation of the cytoplasmic protein NDRG2, indicates that chronic stress induces profound changes in hippocampal glia. Citalopram does not counteract these effects of chronic stress. The cellular mechanisms that are related to the astrocytic changes remain to be elucidated. In addition, the question should be addressed whether such mechanisms protect and support neuronal functioning or whether the stress effects are pathological.
